# *Fructus Ligustri Lucidi* modulates estrogen receptor expression with no uterotrophic effect in ovariectomized rats

**DOI:** 10.1186/s12906-018-2171-3

**Published:** 2018-04-02

**Authors:** Yu-qing Tang, Cheng Li, Xue-jiao Sun, Yi Liu, Xi-ting Wang, Yu-bo Guo, Li-li Wang, Ru-feng Ma, Jian-zhao Niu, Min Fu, Dong-wei Zhang, Yu Li

**Affiliations:** 1 Traditional Chinese Medicine School, Beijing University of Chinese Medicine, Beijing, 100029 People’s Republic of China; 2 Chinese Material Medica School, Beijing University of Chinese Medicine, Beijing, 100029 People’s Republic of China; 3 The Research Institute of McGill University Health Center, Montreal, Quebec H4A 3J1 People’s Republic of China; 4 Diabetes Research Center, Beijing University of Chinese Medicine, Beijing, 100029 People’s Republic of China

**Keywords:** *Fructus Ligustri Lucidi* (FLL), Osteoporosis, Estrogen receptor, Femurs, Tibias, Uteri

## Abstract

**Background:**

Accumulating evidence suggests that *Fructus Ligustri Lucidi* (FLL) plays a beneficial role in preventing the development of osteoporosis. However, the effects of FLL on estrogen receptor (ER) α and ERβ expressions remain unknown. Therefore, in the current study we attempted to probe into the effects of FLL on ERα and ERβ expressions in femurs, tibias and uteri of ovariectomized (OVX) rats.

**Methods:**

The OVX rats were orally administrated with FLL water extract (3.5 g/kg/day) for 12 weeks. The uteri, femurs, tibias and serum were harvested from rats. The serum levels of estrogen (E_2_), luteinizing hormone (LH) and follicle-stimulating hormone (FSH) were determined by ELISA. The expressions of ERα and ERβ in the femurs and tibias as well as uteri were analysed by western blot and immunohistochemical staining.

**Results:**

FLL treatment did not increase uterus relative weight in OVX rats. Further, FLL treatment increased ERα expression in the femurs and tibias, and enhanced ERβ expression in the uteri of OVX rats. However, the resulted expression of ERα was stronger than that of ERβ in OVX rats in response to FLL treatment. Meanwhile, administration with FLL to OVX rats increased FSH and LH but did not increase E_2_ level in the serum.

**Conclusion:**

FLL treatment shows tissue selection on ERα and ERβ expressions in the femurs and tibias as well as uteri of OVX rats without uterotrophic effect, which may offer the scientific evidence of the efficiency and safety of its clinical application.

## Background

As life expectancy around world shows dramatic rise in the last several decades, osteoporosis, one of common chronic metabolic diseases among the elders, has become a prevalent public health problem owing to its high morbidity and mortality [1]. According to the report from International Osteoporosis Foundation, women are at a higher risk of developing osteoporosis than men because of the estrogen deficiency after their menopause [2]. As such, classical hormone replacement therapy (HRT) is widely used to prevent both menopausal symptoms and osteoporotic fractures [3]. Estrogen promotes bone accrual through estrogen receptor (ER) α and ERβ [4]. In addition, deletion of ERα in female mice exhibits reduction of bone mass and strength [5, 6], whereas increased expression of ERα in endothelium is associated with risk of developing breast and uterine cancer, which are also main side effects induced by HRT treatment [7, 8]. Moreover, selective activation of ERβ contributes to inhibition of breast cell proliferation and is also one of optimal targets to elicit beneficial estrogen-like activities [4, 9, 10]. Therefore, discovery and development of selective ER agonist remains a need for osteoporosis treatment.

*Fructus Ligustri Lucidi* (FLL) is the ripe fruit derived from the evergreen tree *Ligustrum lucidum Ait*. It is a common herbal medicine widely used in traditional Chinese medicine (TCM) formula for the management of osteoporosis [11]. FLL extracts have been demonstrated to improve bone quality in diabetic mice [12] and growing female rats [13] through regulation of calcium metabolism via stimulating parathyroid production. We [14] and others [15] also demonstrated that aqueous extracts of FLL improved bone mineral density (BMD) and bone microstructure in ovariectomized (OVX) rats via regulation of collagen metabolism. In addition, FLL ethanol extracts also promote mesenchymal stem cells differentiation [16]. However, little is known about the effect of FLL on ER expression in OVX rats. Therefore, the present study is aimed to explore the effects of FLL on ERα and ERβ expressions in the femurs, tibias and uteri as well as its effects on uterus weight in OVX rats.

## Methods

### Reagents and chemicals

Pentobarbital sodium was purchased from Sigma-Aldrich (St. Louis, USA), Estradiol valerate (EV) tablets were bought from Bayer (Monheim, German). Rat monoclonal anti-ERα antibody (ab3575) and mouse monoclonal anti-ERβ (ab288) were purchased from Abcam (Cambridge, UK). All the other chemicals, except specially identified, were obtained from Beijing Sinopharm Chemical (Beijing, China).

### Preparation of FLL water extracts

FLL was bought from Beijing TongRenTang (Beijing, China) and authenticated by Professor Zexin Ma (TCM museum at Beijing University of Traditional Chinese Medicine (BUCM)). For preparation of FLL water extracts, 100 g of raw FLL was grinded into powder and dissolved in 1000 ml of distilled water by continuous stirring for 48 h under low temperature. Then the aqueous extracts were collected by centrifugation (4000 rpm at 4 °C for 10 min). And the supernatants were harvested and lyophilized to obtain a powder (20 g).

### Animals

Female 12-week-old Sprague Dawley rats (200 ± 20 g) were purchased from Beijing SiBeiFu Animal Technology company (license number: SCXK (Beijing) 2014-0037, Beijing, China). The animals were housed in the clean level conditions (certification number: SCXK (Beijing) 2011-0024) at BUCM with the temperature of 22 ± 1 °C, humidity of 55 ± 5%, and a 12 h-light/dark cycle. All rats had free access to tap water and standard chow. All procedures in this study were approved by the Animal Care Committee of BUCM, Beijing, China.

### OVX rat model establishment

After 1 week of acclimation, the OVX rats were established by removing the bilateral ovaries from the corresponding anesthetized rats. The sham control groups were performed by removing the equal volume of fat surrounding the bilateral ovaries. One week after surgery, the OVX animals were randomly divided into three groups of 9 rats in each, named OVX control, OVX + EV and OVX + FLL, respectively. For the treatment, the rats in the OVX + FLL group were orally administrated with the water extracts of FLL (3.5 g/kg/day). The rats in the OVX + EV group were orally administrated with EV tablets (0.1 mg/kg/day). The rats in OVX control group and Sham control group were orally administrated with the same volume of distilled water.

After 12 weeks of administration, rats were anesthetized by intraperitoneal injection with 1% pentobarbital sodium (0.4 ml/100 g, i.p.). Subsequently, blood was collected from the heart by puncture. Then the rats were sacrificed by cervical dislocation. After that, the uteri, femurs and tibias were harvested for the following experiments.

### Uterus coefficient

After trimming off the fat and absorbing the excess surrounding fluid, the wet weight of the uterus was recorded with analytical balance. Then, the uteri were cut into pieces just above the junction with the cervix. Half of the uteri were stored in liquid nitrogen until use. Another half of it was fixed in 10% neutral buffered formalin for histological analysis. Uterus coefficient was determined by uterus wet weight divided by the corresponding body weight of the rat (g/100 g).

### Estrogen (E2), luteinizing hormone (LH) and follicle-stimulating hormone (FSH)

E_2_, LH and FSH were determined by ELISA (CUSABIO, China) according to the manufacturer’s instructions. All the samples were evaluated in duplicates.

### Immunohistochemical staining

The femurs and uteri were fixed with 10% neutral buffered formalin. Furthermore, the tibias were decalcified in 15% ethylenediaminetetraacetic acid (EDTA) buffer (pH 7.4) for 90 days. After that, the femurs and uteri were dehydrated in graded ethanol, defatted in xylene, and embedded in paraffin. Then, 5 μm sections were deparaffinized in xylene and rehydrated with graded ethanol. Subsequently, the sections were incubated with 3% H_2_O_2_ and antigen retrieval solution (0.1 M sodium citrate buffer, pH 6.0) followed by incubation with 10% goat serum in phosphate-buffered saline (PBS) for 30 min to block nonspecific binding sites. The sections were then incubated with the primary antibodies (anti-ERα antibody (1:500) or anti-ERβ antibody (1:500)) overnight at 4 °C. The next day, after washing in PBS, the sections were incubated with biotinylated anti-rat secondary antibody for 30 min and with peroxidase for 10 min according to the SP staining system (Beijing ZhongShan JinQiao, Beijing, China). Diaminobenzidine (DAB) was used as the substrate for color development and visualization under the microscope. For controls, the primary antibodies were replaced by non-immunized goat serum. The slides were then taken for histopathological evaluations. The results of immunohistochemical staining were quantified by Image Pro-Plus software (version 6, SPSS Inc., Chicago, IL, USA) and the integral optical density (IOD) values were recorded. The measurements were performed by two investigators who were blinded regarding the animals’ treatment groups.

### Western blot analysis

The uteri were placed in a 1.5 ml Eppendorf tube and washed with PBS twice, and then were cut with scissors and grounded. The tibias were prepared by lyophilizing and grinding. After that, the samples were lysed in a buffer containing 20 mM Tris–HCl, pH 7.5, 0.1% (*v*/v) Igepal, 6 mM sodium deoxycholate, 150 mM NaCl, 2 mM ethyleneglycoltetraacetic acid (EGTA), 2 mM EDTA, 0.1 mM Na_2_SO_4_, 20 mM NaF, and a protease inhibitor cocktail tablet (Roche, German). The lysates were centrifuged at 10,000 g for 15 min at 4 °C, and protein concentrations in the supernatants were determined by BCA protein assay kit (Applygene, China). Then 50 μg/lane of proteins were loaded into 10% polyacrylamide gel, and transferred onto nitrocellulose membrane, and then incubated with the primary antibody (anti-ERα or anti-ERβ) and the corresponding HRP labeled secondary antibody. The membranes were developed using enhanced chemiluminescence solution. The images were captured with Bio-Rad bioimaging system. The gray values of the blots were quantified using the Image J software (NIH, Bethesda, MD), and normalized with the corresponding β-actin (1:2000) as the internal control.

### Statistical analysis

Data were expressed as the mean ± standard deviation (SD). One-way analysis of variance (ANOVA) was performed between multiple groups using SPSS software (Version 20.0) when homogeneity of variance and normality were met. Otherwise, Dunnett’s T3 and Nonparametric tests were conducted between multiple groups, respectively. *P* values less than 0.05 were considered to be statistically significant.

## Results

### Effects of FLL on the alterations of uterus coefficient in OVX rats

The uterus coefficient of the rats were shown in Table 1. As expected, ovariectomy resulted in a significant reduction in the relative uterus weight of the rats. The uterus coefficient in the OVX control group was only around 16% of that in the Sham control group. EV treatment for 12 weeks significantly increased the uterus coefficient in OVX rats (*P* < 0.05). By contrast, FLL treatment did not increase the uterus coefficient in OVX rats.

**Table 1:** The uterus coefficient in the different groups of rats

Groups	Number	Uterus weight/Body weight (g/100 g)
Sham	9	0.2132 ± 0.0312^*^
OVX	9	0.0357 ± 0.0143
OVX + EV	9	0.1493 ± 0.0240^*^
OVX + FLL	9	0.0322 ± 0.0137

*Compared with OVX group rats, *P < 0.05

### Effects of FLL on E_2,_ LH and FSH levels in serum

As shown in Table 2, serum E_2_ level was decreased, and serum LH and FSH levels were increased in the OVX control group rats as compared to those of rats in the Sham control group. The administration of EV and FLL to OVX rats for 12 weeks significantly decreased serum LH and FSH levels (*P* < 0.05). However, FLL treatment did not increase serum E_2_ in OVX rats (*P* > 0.05).

**Table 2:** Serum levels of E_2_, LH and FSH in the different groups of rats

Groups	Number	E_2_ (pg/ml)	LH (mUI/ml)	FSH (mUI/ml)
Sham	9	15.5975 ± 5.0579^*^	2.1711 ± 0.8571^*^	0.8411 ± 0.3071^*^
OVX	9	6.65 ± 2.3853	3.8744 ± 0.8100	1.8837 ± 0.3301
OVX + EV	9	11.9755 ± 1.0535^*^	2.5850 ± 0.4829^*^	1.2087 ± 0.1795^*^
OVX + FLL	9	6.8842 ± 1.9301	3.0455 ± 0.5554^*^	1.4512 ± 0.2801^*^

*Compared with OVX group rats, *P < 0.05

### Effects of FLL on the expressions of ERα and ERβ in the femurs and tibias of OVX rats

**Fig. 1:**
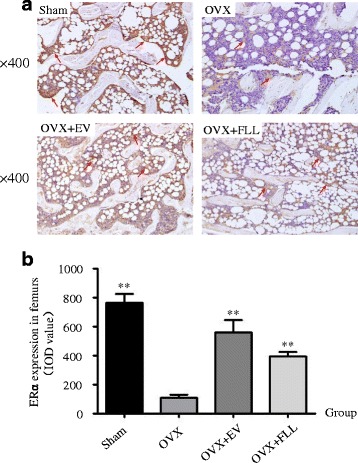
The representative images (**a**) and images analysis results of immunohistochemical staining (**b**) showing the effect of FLL on ERα expression in the femurs of the rats. The arrows illustrated the expression and distribution of ERα. Data are presented as mean ± SD. **P < 0.01 compared with the OVX control group

**Fig. 2:**
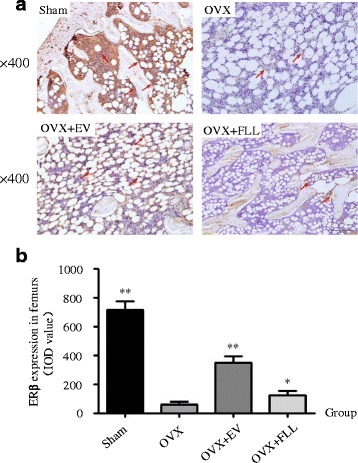
The representative images (**a**) and images analysis results of immunohistochemical staining (**b**) showing the effect of FLL on ERβ expression in the femurs of the rats. The arrows illustrated the expression and distribution of ERβ. Data are presented as mean ± SD. *P < 0.05 or **P < 0.01 compared with the OVX control group

The effects of FLL on ERα and ERβ expressions in the femurs were assessed by immunohistochemical staining. As shown in Figs. 1 and 2, ERα and ERβ expressions in the femurs of the OVX control group were significantly decreased (*P* < 0.01), when compared with those of rats in the Sham control group. Both FLL and EV treatment significantly increased ERα and ERβ expressions in the femurs of the OVX rats (*P* < 0.05 or 0.01) when compared to those in the OVX control group.

**Fig. 3:**
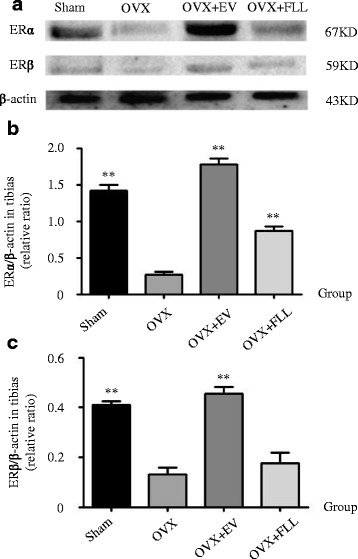
The representative images (**a**) and images analysis results of western blot (**b** and **c**) showing the effects of FLL on the expressions of ERα and ERβ in the tibias of the rats. β-actin was taken as the internal control. Data are presented as mean ± SD. **P < 0.01 compared with the OVX control group

Furthermore, the effects of FLL on ERα and ERβ expressions in the tibias of the rats in different groups were also evaluated by western blot. As shown in Fig. 3, FLL treatment did markedly increase ERα expression in response to ovariectomy (*P* < 0.01). By contrast, FLL treatment showed a trend toward increasing ERβ expression in OVX rats, but the differences did not reach the statistically significant level when compared to those of rats in the OVX control group.

### Effects of FLL on the expressions of ERα and ERβ in the uteri of OVX rats

**Fig. 4:**
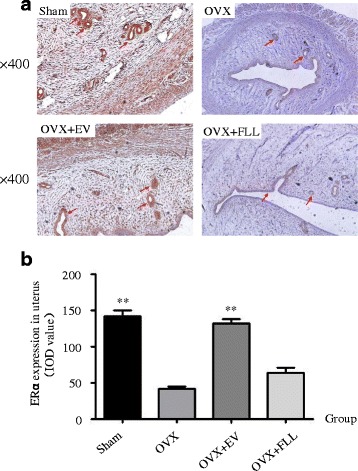
The representative images (**a**) and images analysis results of immunohistochemical staining (**b**) showing the effect of FLL on ERα expression in the uteri of the rats. The arrows illustrated the expression and distribution of ERα. Data are presented as mean ± SD. **P < 0.01 compared with the OVX control group

**Fig. 5:**
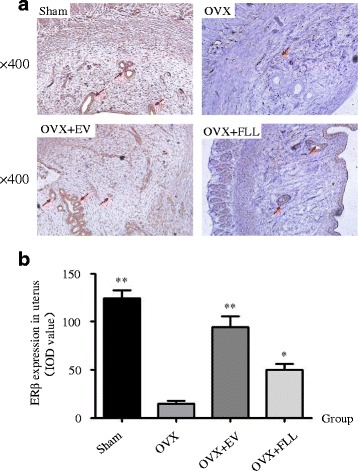
The representative images (**a**) and images analysis results of immunohistochemical staining (**b**) showing the effect of FLL on ERβ expression in the uteri of the rats. The arrows illustrated the expression and distribution of ERβ. Data are presented as mean ± SD. *P < 0.05 or **P < 0.01 compared with the OVX control group

**Fig. 6:**
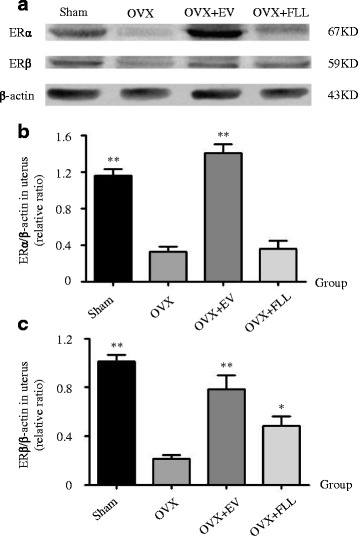
The representative images (**a**) and images analysis results of western blot (**b** and **c**) showing the effects of FLL on the expressions of ERα and ERβ in the uteri of the rats. β-actin was taken as the internal control. Data are presented as mean ± SD. *P < 0.05 or **P < 0.01 compared with the OVX control group

As shown in Figs. 4 and 5, ERα and ERβ were mainly located in the endometrium and glandular epithelia cells, and were highly expressed in the uteri of rats in sham control group and EV treatment group compared to those of rats in the OVX control group as evaluated by immunohistochemical staining (*P* < 0.01). Further, FLL treatment did not increase ERα expression but did obviously increase ERβ expression in the uteri of the OVX rats (*P* < 0.05). These results were also further confirmed by western blot (Fig. 6).

## Discussion

In the current study, we demonstrated that FLL treatment decreased serum LH and FSH levels but not increased serum E_2_ level in OVX rats. FLL did not increase uterus relative weight in response to ovarirectomy. In addition, FLL treatment significantly enhanced ERβ expression, but left no evident influence on ERα in uteri. Moreover, FLL treatment markedly enhanced ERα expression, but had no obvious effect on ERβ expression in the femurs and tibias. These results suggest that FLL may show different effects on ER expression in bones and uteri, which may contribute to uterus health during the treatment of osteoporosis.

Both ERα and ERβ are the main targets of estrogen and can be either coexistent or expressed alone in various tissues [17]. ERα is inclined to be found in the ovary, breast, hypophysis, paranephros, kidney and bone, while ERβ is highly concentrated in the granulosa cells of prostate and ovary, commonly expressed in ovary, lung, brain and testis, and less expressed in hypophysis and spinal cord [18, 19]. In the reproductive system, ERα expression decreases gradually from the epithelial cells of vagina to those of oviduct [20, 21]. In the uteri of OVX rats, the expression of ERβ are tremendously reduced and almost only ERα can be detected [22, 23]. Over-expression of ERα may lead to the hyperplasia of mammary glands and endometrial cells, and result in an increased risk of breast cancer and endometrial cancer [24]. In contrast, activation of ERβ never causes the relevant cell proliferation, instead, it has certain effects against cell proliferation [25]. In the current study, we found that FLL treatment did increase ERβ expression in the uteri though with no significant effect on ERα expression, which may contribute to maintain the uterus health during the management of osteoporosis. The results suggest that FLL may selectively enhance ERβ expression in uteri of OVX rats. As compared to other phytoestrogens with high affinities with ERα, the application of FLL does not increase the risk of endometrial hyperplasia.

ERα and ERβ possess vast expressions and may antagonize each other in the bones and bone marrows [26, 27]. Deletion of ERα in osteoblast or osteoclast impedes bone formation and strength accrual in female mice [28]. Inhibition of ERα (not ERβ) expression in osteoclast promotes bone resorbing activity [29]. Deletion of ERα in osteoclast attenuates protective effect of estrogen on cancellous bone through increasing osteoclastogenesis. Furthermore, double deficiency of ERα and ERβ has similar reductions in BMD versus single deficiency of ERα in mice [30]. The results form clinical trials also support the notion that ERα (not ERβ) plays a beneficial role in maintaining bone health in men [31]. In addition, the advantage of ERα over ERβ favors for bone formation and fracture healing in OVX rats [26]. These findings suggest that the protective effect of estrogen on BMD in trabecula may be practiced via ERα. In conjunction with our current finding of an increase in ERα expression in rats of FLL treatment group and the results from our previous publication [14] as well as other groups [15, 30, 32], the results indicate that FLL treatment may boost bone density through induction of ERα expression in OVX rats.

ERα and ERβ may play different roles in the bone metabolism and remodeling. These roles are different in some aspects yet interrelated in other aspects. Estrogen’s effect on bones is likely to function through the mediation of ERα. The two subtypes of ER may exist as a complementary relationship, in other words, when ERα exists, ERβ may weaken the transcriptional activation function of ERα, and while ERα is absent, ERβ can partially replace some functions of ERα. Selective agonist of ERα can entirely offset the influence of ovariectomy on uterus weight and BMD in the experimental rats [7, 19]. The findings of our current study here also show that aqueous extract of FLL could enhance ERα expression, and have no apparent influence on ERβ expression in the femurs and tibias of OVX rats.

In the current study, we found that FLL treatment significantly decreased the levels of LH and FSH but did not increase E_2_ levels in OVX rats. Ovariectomy in rats results in a decrease in E_2_ and an increase in LH and FSH [33, 34]. Deficiency of E_2_ significantly promotes bone loss and aggravates uterus atrophy [34, 35]. Inhibition of FSH also impairs bone loss and further prevents LH release while the alteration does not play a dominant role in the development of osteoporosis [35]. In addition, high circulating FSH contributes to endometrial atrophy in mice [36]. Increased circulating LH may be associated with postmenopausal “hot flushes” [37]. The increase of FSH may contribute to uterus atrophy. The results suggest that FLL could alleviate postmenopausal vasomotor symptoms, which required further investigation.

In addition, we have demonstrated that FLL water extract mainly includes salidroside, ligustroflavon, acteoside, specnuezhenide, and oleuropein acid [38]. Currently, salidroside was demonstrated to bind to ERα in docking simulation assay [39]. So it is reasonable to deduce that salidroside may account for the estrogen-like effect of FLL in OVX rats. However, further studies are still needed to identify the contributions of each component in the FLL aqueous extract.

## Conclusion

In conclusion, FLL treatment increases ERβ expression in uteri and strengthens ERα expression in the femurs and tibias as well as poses no risk of the increasing of uterus relative weight in OVX rats. In addition, our findings also demonstrate that FLL has the ability of coordinating LH and FSH levels in circulation, which may contribute to alleviate postmenopausal vasomotor symptoms. However, how FLL regulates ER expression in OVX rats still needs further investigation.

## Abbreviations

BMD: Bone mineral density
BUCM: Beijing University of Traditional Chinese Medicine
DAB: Diaminobenzidine
E_2_: Estrogen
EDTA: Ethylenediaminetetraacetic acid
EGTA: Ethyleneglycoltetraacetic acid
ER: Estrogen receptor
EV: Estradiol valerate
FLL: *Fructus Ligustri Lucidi*
FSH: Follicle-stimulating hormone
HRT: Hormone replacement therapy
IOD: Integral optical density
LH: Luteinizing hormone
OVX: Ovariectomized
PBS: Phosphate-buffered saline
TCM: Traditional Chinese medicine
